# Evidence-Based Data Models for Return-to-Play Criteria after Anterior Cruciate Ligament Reconstruction

**DOI:** 10.3390/healthcare10050929

**Published:** 2022-05-18

**Authors:** Matthew C. Daggett, Kevin A. Witte, Dimitrije Cabarkapa, Damjana V. Cabarkapa, Andrew C. Fry

**Affiliations:** 1 Sano Orthopedics, Lee’s Summit, MO 64064, USA; mdaggett@sanoorthopedics.com (M.C.D.); kwitte@sanoorthopedics.com (K.A.W.); 2 Jayhawk Athletic Performance Laboratory, Wu Tsai Human Performance Alliance, University of Kansas, Lawrence, KS 66045, USA; d927c184@ku.edu (D.V.C.); acfry@ku.edu (A.C.F.)

**Keywords:** knee, sport, mobility, alignment, readiness, injury, sport performance, exercise, reinjury

## Abstract

The anterior cruciate ligament (ACL) tear is one of the most common knee injuries in sports that require side-to-side pivoting movements. While the timeline and specific goals during rehabilitation protocols may vary, ACL reconstruction (ACLR) is the preferred procedure necessary to return these athletes to their respective field of play. However, there are no validated guidelines that define a specific timepoint when it is safe for an athlete to return-to-play, as functional movement deficit may be present much longer than six months post ACLR. A retrospective cross-sectional analysis was conducted on 33 subjects that underwent ACLR. As a part of standard of care, each subject completed a movement screening protocol at a singular timepoint during their rehabilitation process. An innovative three-dimensional markerless motion capture system was used to obtain three algorithm-derived biometric variables: mobility, alignment, and readiness. Significant gradual improvements in mobility and readiness were observed throughout a 3–6-month post ACLR procedure period. When examining the data trends, it was obvious that not all patients responded identically to treatment plans provided by clinical professionals. Therefore, the findings of the present study suggest that the decision regarding when it is safe to return to play needs to be determined on an individual basis.

## 1. Introduction

The anterior cruciate ligament (ACL) is one of the key anatomical structures that aids in stabilizing the knee joint. Despite many health-related benefits associated with participation in sport, the knee is one of the most injury prone joints due to its central role in supporting large and rapidly changing external loads during various types of sport-specific activities [1]. An ACL tear is one of the most common knee injuries in sports that require side-to-side pivoting movements such as basketball, American football, and volleyball [2,3]. While the timeline and specific goals during rehabilitation protocols may vary, it is currently believed that ACL reconstruction (ACLR) is the preferred surgical procedure necessary to return these athletes to their respective field of play [2]. There are approximately 350,000 ACLR procedures performed annually in the Unites States, and approximately one million worldwide [4,5]. However, the prevalence of ACL re-rupture rates in young and active adults is up to 20%, with 50% of them occurring during the first year after the primary ACLR procedure [6,7]. This may be mainly attributed to premature return to sports, as 82.6% of patients approximately eight months post ACLR had functional deficit in one or more return-to-sport screening parameters when compared with their age- and sex-matched peers [6]. Interestingly, although confident young athletes were more likely to meet return-to-sport criteria following ACLR they were concurrently more likely to sustain second ACL injury [8].

What is clear regarding this research topic is that there are no validated guidelines that define a specific timepoint when it is safe for an athlete to return-to-play [9], as functional movement deficit may be present much longer than six months post ACLR [1,10]. Goerger et al. [11] found that alterations in movement patterns did not resolve even after the completion of a rehabilitation program, whereas deficits in quadriceps strength and rate of force development persisted beyond six months post ACLR and return to participation in sport-specific activities [12,13]. Based on these findings, it seems that patients may be able to mask their functional deficiencies by adopting super-compensatory movement patterns in areas not specific to their surgical location. In addition, it has been shown that 35% of athletes do not return to their preinjury level of performance within two years post ACLR [1,14]. Therefore, without well established guidelines and without device-based objective performance assessment methodologies, patient’s improvements can be challenging to monitor for a qualified health professional and the determination of return-to-play criteria can be confounded.

The purpose of the present study was to retrospectively examine post ACLR return-to-play progress based on three algorithm-derived functional movement screening scores (i.e., mobility, alignment, readiness) obtained from an innovative three-dimensional markerless motion capture system as an objective performance assessment modality.

## 2. Materials and Methods

### 2.1. Procedures

Thirty-three subjects were included in this retrospective cross-sectional analysis. The following inclusion criteria were used: (i) aged between 15 and 25 years old; (ii) sustained unilateral ACL injury; (iii) underwent ACLR, (iv) completed a standardized rehabilitation protocol. The following exclusion criteria were used: (i) sustained contralateral ACL tear; sustained bilateral ACL tear; (iii) had previous knee surgery. As a part of standard of care, each subject completed biomechanical testing at a singular time point during their six-month long rehabilitation procedure. The testing protocol consisted of nine body movements performed in sequential order. The detailed description of each movement incorporated into the screening protocol is presented in Table 1. An innovative three-dimensional markerless motion capture system (DARI Motion, Overland Park, KS, USA), cleared by the Food and Drug Administration (FDA), was used to obtain biomechanical parameters (i.e., kinetics and kinematics) of each body motion. This system is composed of eight high-definition cameras sampling at 60 Hz. The cameras are positioned at different orientations to completely surround and cover the testing area. The visual hull technology model records and subtracts the visual signal minus the background, which is being used to generate a pixelated person to obtain biomechanical parameters of each body movement. Moreover, the confidence in the test measurement validity and reliability of three-dimensional markerless motion capture systems for assessment of functional movement parameters has been successfully investigated [15,16,17,18,19,20].

Prior to each testing session, the system calibration was performed following manufacturer-based recommendations. For the purpose of consistency, a trained research team member, standing outside of the camera capture area, provided the subject with the following command: “one, two, three, begin”. At the command “begin“, the subject performed the designated movement while the motion was being simultaneously recorded by the three-dimensional markerless motion capture system. After completion of the movement, the subject received the command “done”, after which the recording was stopped. This procedure was repeated for each of the nine motions separately. After the completion of the testing protocol, the data was automatically processed and analyzed by DARI Motion defined algorithms to derive three unique biometric ratings: mobility, alignment, and readiness. The approximate time for completion of all data analysis procedures was approximately 30–60 s.

Mobility and alignment biometric ratings represent knee joint range of motion in the primary (flexion/extension) and non-primary (varus/valgus) planes of movement, respectively. For each motion, peak knee flexion and valgus values were calculated (degrees) and compared with a population range for that specific movement. A DARI Motion database consisting of more than 10,000 individuals (15–80 years old) who have completed identical movements was used to determine normative ranges for each variable. After going through the data normalization process, the knee flexion and valgus values were converted to percentile rankings and averaged to derive mobility and alignment biometric ratings. In addition, readiness is an aggregate biometric that utilizes mobility and alignment ratings across all body motions to generate an overall performance measure. For each movement, joint specific functionality and center of motion performance parameters were obtained and normalized based on subject’s anthropometric characteristics. All performance measurements for each individual movement were combined and averaged to create a readiness score as a single metric that represents overall ability of an individual to complete a specific movement protocol.

### 2.2. Statistical Analysis

Descriptive statistics, means and standard deviations ($\overset{-}{x}$ ± SD), were calculated for each dependent variable. A second-order polynomial regression (i.e., quadratic fit) was used to examine the relationship between algorithm-derived biometric scores (i.e., mobility, alignment, readiness) throughout a 3–6-month post ACLR procedure period. The normality of the data was assessed using the Shapiro–Wilk test. Alignment and readiness biometric ratings showed normal distribution, whereas mobility was not normally distributed. A one-way analysis of variance (ANOVA) with Bonferroni post hoc adjustments was used to test for statistically significant changes in alignment and readiness scores between each month post ACLR. The Kruskal–Wallis test and a post hoc Mann–Whitney U-test with Bonferroni adjustments was used to test for statistically significant changes in mobility scores between each month post ACLR. Statistical significance was set a priori to *p* < 0.05. All statistical analyses were completed with SPSS (Version 26.0; IBM Corp., Armonk, NY, USA) and Microsoft Excel (Microsoft Corp., Redmond, WA, USA).

## 3. Results

**Figure 1:**
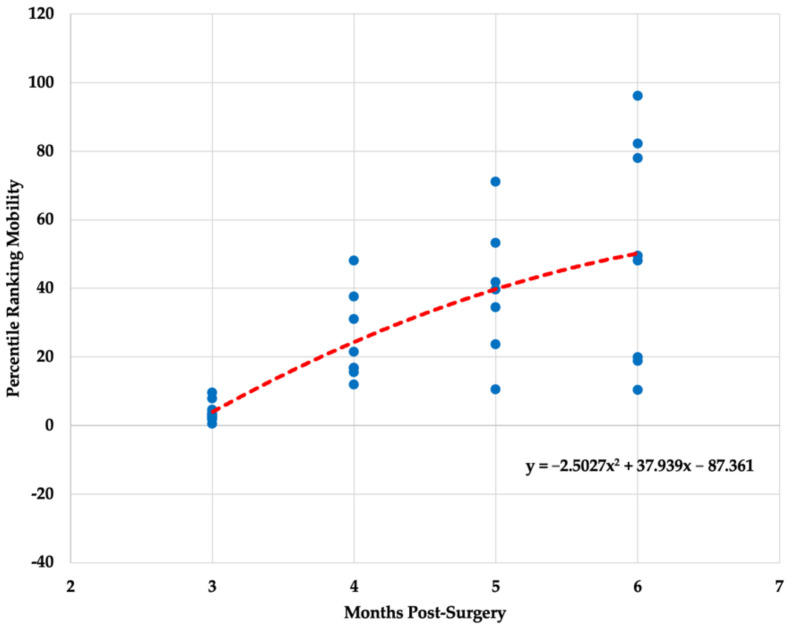
Polynomial regression model for mobility variable.

**Figure 2:**
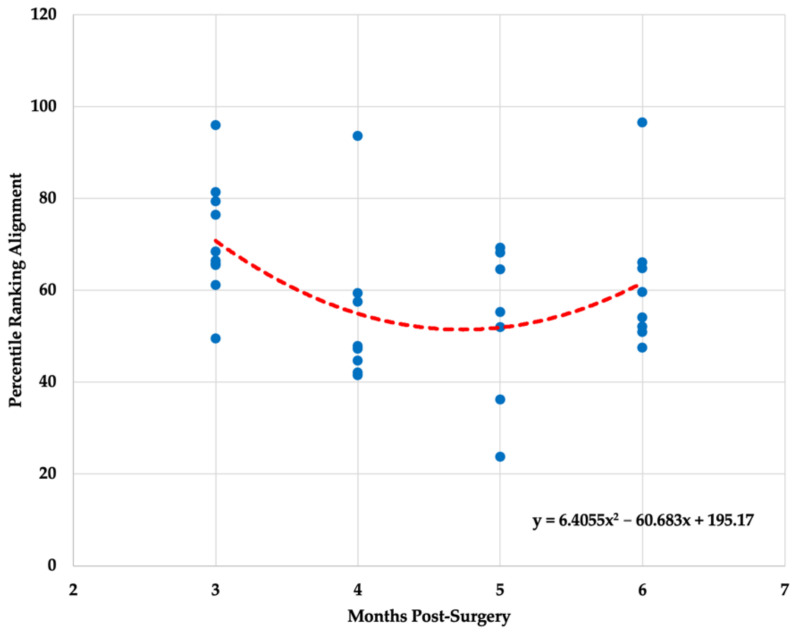
Polynomial regression model for alignment variable.

**Figure 3:**
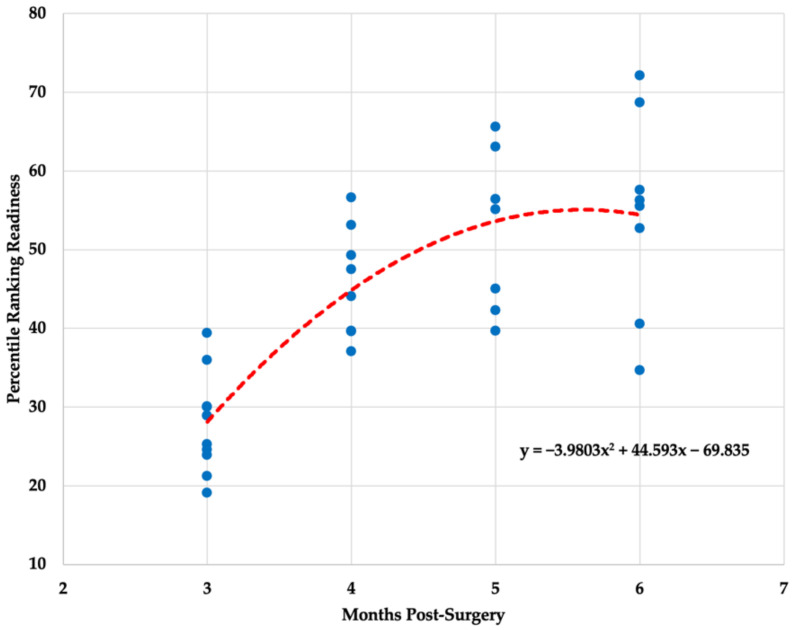
Polynomial regression model for readiness variable.

Descriptive statistics ($\overset{-}{x}$ ± SD) for each biometric variable (i.e., mobility, alignment, readiness) is presented in Table 2. All three polynomial regression models for mobility (F _(2,30)_ = 14.825, *p* < 0.001, R^2^= 0.497), alignment (F_(2,30)_ = 3.898, *p* = 0.031, R^2^ = 0.206) and readiness (F_(2,30)_ = 24.611, *p* < 0.001, R^2^ = 0.621) ratings were statistically significant. The graphical representation of the results is presented in Figure 1, Figure 2 and Figure 3.

The overall ANOVA model was statistically significant for the readiness variable (F_(3,29)_ = 16.065, *p* < 0.001) and non-statistically significant for the alignment variable (F_(3,29)_ = 2.529, *p* < 0.077). Readiness scores were significantly different between the third and fourth (*p* = 0.002), third and fifth (*p* < 0.001), and third and sixth month (*p* < 0.001) post ACLR. A Kruskal–Wallis test revealed a statistically significant changes in mobility scores across multiple months post ACLR (H_(2)_ = 21.11, *p* < 0.001). Mobility scores were significantly different between the third and fourth (*p* = 0.030), third and fifth (*p* = 0.001), and third and sixth month (*p* < 0.001) post ACLR.

## 4. Discussion

The findings of the present study indicate significant gradual improvements in mobility and readiness scores throughout a 3–6-month post ACLR procedure period. However, when looking at the data trends, it is obvious that not all patients responded identically to treatment plans provided by clinical professionals. The quality of the movement that the patient demonstrates post ACLR is of critical importance, if not even more important than quantitative measures commonly used to establish return-to-play criteria [21]. Most patients seem to start at a similar level approximately three months post ACLR, but their progressions tend to deviate as the rehabilitation process continues. Some patients may progress quickly, whereas others may take more time to reach the same level of movement capabilities. In terms of neuromuscular abilities, most patents might not be ready for safe return-to-play even after eight months post ACLR [6]. Therefore, it is evident that patient care post ACLR and the decision regarding when it is safe to return-to-play needs to be determined on an individual basis.

The regression models established in the present study for the three algorithm-derived biometric variables obtained from the three-dimensional markerless motion capture system may be used to track a patient’s recovery progression post ACLR. However, it is important to note that the effectiveness of the rehabilitation process and the overall patient’s progression should not be determined by tracking a single biometric variable (e.g., readiness). Previous research has indicated the presence of biomechanical alterations across various sport-specific motions (e.g., jumping, landing, sidestep cutting) in patents that underwent ACLR procedure as an overloading protective mechanism [10,22]. A 14% decrease in total movement and 35% decrease in total power was observed in an injured limb when compared with an uninjured limb [23]. Jump height, knee-joint extension, and ankle plantar flexion were also notably lower for an injured limb post ACLR [23]. In addition, Orishimo et al. [22] found that decreased power production during takeoff in the injured limb was primarily compensated by higher power produced at the hip joint, whereas greater power absorption at the ankle joint was acting as a super-compensatory mechanism for a decreased power absorption at the knee joint during landing maneuvers. Thus, improvements in overall readiness scores should be interpreted in conjunction with mobility and alignment scores to assure that the observed improvements were not attained by super-compensatory mechanisms such as changes in hip and ankle biomechanical parameters.

Although not reaching the level of statistical significance, the alignment scores demonstrated a progressive downward trend through the initial phase of the ACLR recovery period, which may be attributed to the improvements in the overall knee mobility. This information may allow clinical practitioners to obtain a deeper insight into areas for further improvements that should be simultaneously targeted to design a more complete recovery plan. Another clinically relevant point is that tracking a patient’s progress via non-invasive three-dimensional markerless motion capture technology can aid with an objective decision-making process. In a majority of therapy programs, patients are expected to complete exercises on their own and return for evaluation of their progress every two weeks. At six months post operatively, both patient and health practitioner should have enough information to determine their release from care. Previous research has indicated that incorporating innovative device-based assessment modalities in a complementary manner with some of the traditional assessment approaches could optimize return-to-play or meeting physical activity guideline levels post ACLR [24]. However, without a medical device for tracking a human motion such as the one used in the present investigation, the review of a patient’s progress remains predominantly subjective. Greater testing frequency (e.g., every 2–3 weeks) may also assist clinical practitioners with the decision-making process if a patient is ready to transition into a new phase of the rehabilitation program. For example, performing plyometric exercises, lunges, and single leg squats should not occur without mastering some of the early goals of the initial phase of the rehabilitation process such as achieving an appropriate level of knee joint range of motion (i.e., full knee extension and at least 90 degrees of flexion) [2]. Additionally, an increase in testing/screening frequency may lead to the improvements in patent’s compliance within and outside of the rehabilitation setting, which has shown to be one of the critical factors related to achieving the desired return-to-play timeline [2]. Therefore, by utilizing non-invasive technology for human motion assessment to obtain metrics such as those shown in the present study, health providers can gain additional information necessary for determining an optimal return-to-play timeline on an individual basis that could potentially minimize risk for reinjury.

## Figures and Tables

**Table 1:** List and description of body movements incorporated into screening protocol.

Specific Body Movement	Description of Movement
Bilateral Squat	Start with feet forward and shoulder-distance apart, and while holding a light bar directly above the head, lower body downward as far as possible.
Right Leg (Unilateral) Squat	Start by raising left foot off the ground and while balancing on the right leg, lower body down as far as possible on the standing leg and return to the starting position.
Left Leg (Unilateral) Squat	Start by raising right foot off the ground and while balancing on the left leg, lower body down as far as possible on the standing leg and return to the starting position.
Right Leg Lateral Lunge	Start by taking two large steps to your left within the capture space. Push off with your left leg and bound as far to your right side. Land on your right leg and immediately push off in the opposite direction to reach the starting position.
Left Leg Lateral Lunge	Start by taking two large steps to your right within the capture space. Push off with your right leg and bound as far to your left side. Land on your left leg and immediately push off in the opposite direction to reach the starting position.
Right Unilateral Jump	Start with feet forward, legs straight and arms extended backwards as far as possible, raise left leg off ground then jump as high as possible off right leg.
Left Unilateral Jump	Start with feet forward, legs straight and arms extended backwards as far as possible, raise right leg off ground then jump as high as possible off left leg.
Five Hops Right Leg	Start with feet forward, lift left leg to a near 90-degree angle, then jump as high as possible off right leg, five consecutive times.
Five Hops Left Leg	Start with feet forward, right left leg to a near 90-degree angle, then jump as high as possible off left leg, five consecutive times.

**Table 2:** Descriptive statistics ($\overset{-}{x}$ ± SD) and coefficient of variation percentage (CV%) for each biometric variable obtained from an innovative three-dimensional markerless motion capture system.

Time Post ACLR	Mobility (%)	Alignment (%)	Readiness (%)
Three Months	3.79 ± 2.81 (74.02)	70.96 ± 12.80 (18.04)	27.85 ± 6.36 (22.84)
Four Months	24.86 ± 12.75 * (51.29)	54.20 ± 17.23 (31.79)	45.88 ± 6.96 * (15.17)
Five Months	39.19 ± 19.59 * (49.98)	52.72 ± 17.18 (32.58)	52.46 ± 10.25 * (19.53)
Six Months	50.43 ± 32.53 * (64.61)	61.42 ± 15.65 (25.47)	54.78 ± 12.61 * (23.02)

Note: * significantly different when compared with three months post ACLR (*p* < 0.05).

## Data Availability

The data sets associated with the findings of the present study are available from the corresponding author upon reasonable request.
